# GEP analysis validates high risk MDS and acute myeloid leukemia post MDS mice models and highlights novel dysregulated pathways

**DOI:** 10.1186/s13045-016-0235-8

**Published:** 2016-01-27

**Authors:** Laura Guerenne, Stéphanie Beurlet, Mohamed Said, Petra Gorombei, Carole Le Pogam, Fabien Guidez, Pierre de la Grange, Nader Omidvar, Valérie Vanneaux, Ken Mills, Ghulam J Mufti, Laure Sarda-Mantel, Maria Elena Noguera, Marika Pla, Pierre Fenaux, Rose Ann Padua, Christine Chomienne, Patricia Krief

**Affiliations:** Université Paris-Diderot, Sorbonne Paris Cité, Institut Universitaire d’Hématologie, Unité Mixte de Recherche (UMR-S) 1131, Paris, France; Institut National de la Santé et de la Recherche Médicale (INSERM) Unité (U) 1131, Paris, France; Department of Haematological Medicine, King’s College London and Kings College Hospital, London, UK; GenoSplice technology, iPEPS-ICM, Hôpital de la Pitié Salpêtrière, Paris, France; Haematology Department, Cardiff University School of Medicine, Cardiff, UK; Assistance Publique-Hôpitaux de Paris (AP-HP), Unité de Thérapie Cellulaire, Hôpital Saint Louis, Paris, France; Centre for Cancer Research and Cell Biology, Queen’s University Belfast, Belfast, UK; Université Paris-Diderot, Sorbonne Paris Cité, Institut Universitaire d’Hématologie Hôpital Saint Louis, Paris, France; Assistance Publique-Hôpitaux de Paris (AP-HP), Service de Médecine Nucléaire, Hôpital Lariboisière, Paris, France; Assistance Publique-Hôpitaux de Paris (AP-HP), Laboratoire d’Hématologie, Hôpital Saint Louis, Paris, France; Université Paris-Diderot, Sorbonne Paris Cité, Département d’Expérimentation Animale, Institut Universitaire d’Hématologie, Paris, France

**Keywords:** Myelodysplastic syndrome, Mice models, Gene expression profile, MDS

## Abstract

**Background:**

In spite of the recent discovery of genetic mutations in most myelodysplasic (MDS) patients, the pathophysiology of these disorders still remains poorly understood, and only few in vivo models are available to help unravel the disease.

**Methods:**

We performed global specific gene expression profiling and functional pathway analysis in purified Sca1+ cells of two MDS transgenic mouse models that mimic human high-risk MDS (HR-MDS) and acute myeloid leukemia (AML) post MDS, with NRASD12 and BCL2 transgenes under the control of different promoters _MRP8_NRASD12/_tet_hBCL-2 or _MRP8_[NRASD12/hBCL-2], respectively.

**Results:**

Analysis of dysregulated genes that were unique to the diseased HR-MDS and AML post MDS mice and not their founder mice pointed first to pathways that had previously been reported in MDS patients, including DNA replication/damage/repair, cell cycle, apoptosis, immune responses, and canonical Wnt pathways, further validating these models at the gene expression level. Interestingly, pathways not previously reported in MDS were discovered. These included dysregulated genes of noncanonical Wnt pathways and energy and lipid metabolisms. These dysregulated genes were not only confirmed in a different independent set of BM and spleen Sca1+ cells from the MDS mice but also in MDS CD34+ BM patient samples.

**Conclusions:**

These two MDS models may thus provide useful preclinical models to target pathways previously identified in MDS patients and to unravel novel pathways highlighted by this study.

**Electronic supplementary material:**

The online version of this article (doi:10.1186/s13045-016-0235-8) contains supplementary material, which is available to authorized users.

## Background

Myelodysplastic syndrome (MDS) is an hematopoietic stem cell disorder resulting in aberrant cell growth and differentiation with enhanced genomic instability. The diagnosis of MDS is based on morphological features (dysplasia) of the blood and bone marrow (BM) cells, cytopenias, frequent excess of marrow blasts, and specific cytogenetic abnormalities [1] such as deletions of chromosome 5, 7, and trisomy 8. Transformation to acute myeloid leukemia (AML) occurs in up to 40 % of MDS patients [2]. Major advances have been acquired in the understanding of MDS pathogenesis. Along with refined risk stratification scores combining clinical and cytogenetic abnormalities, whole-genome sequencing (WGS) and next-generation sequencing (NGS) have identified mutations in genes involved in signal transduction, splicing machinery, and epigenetic or transcriptional pathways [3–11]. Gene expression profiling is a comprehensive approach taking into account the majority of these altered pathways and has contributed to a better definition of diagnosis and prognosis in many diseases including MDS, when combined with gene mutation or methylation data. [12–24]. Nevertheless, the survival of MDS patients, namely with high-risk MDS remains extremely poor and therapy options scarce.

Animal models expressing MDS associated gene phenocopy either the MDS disease or more often its risk of transformation to AML [25–27]. They have been instrumental to tackle other aspects of the disease such as the involvement of the microenvironment [28], and we have shown how they can be used to point at pathways to identify novel biomarkers and targeted therapies [29–35].

We therefore analyzed the gene expression pathways and ontology functional groups in Sca1+ cells from two MDS mouse models we have previously described [29, 30, 32–35].

**Fig. 1:**
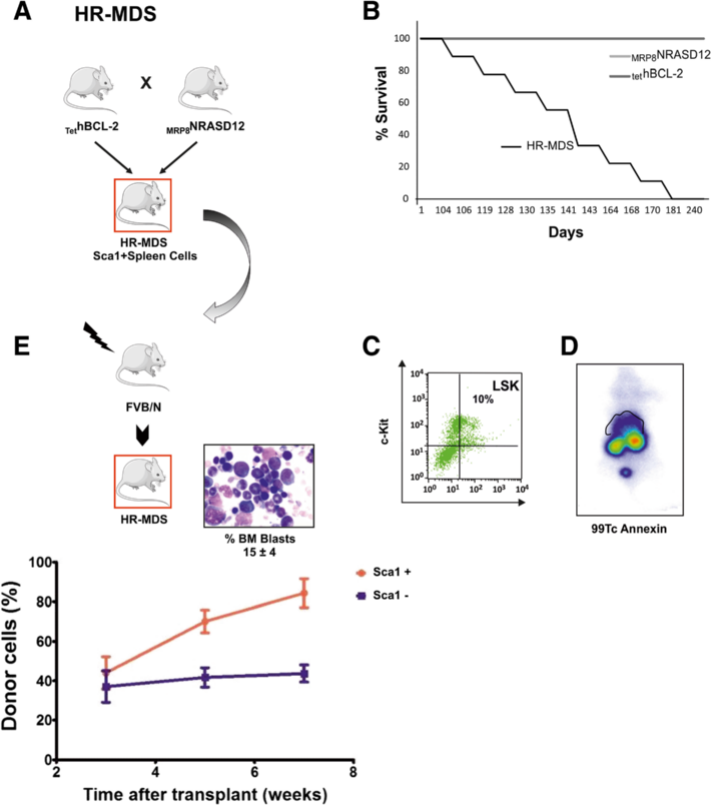
**a** Schematic representation of the HR-MDS mouse model. The characteristics of this model have previously been published ^[24-26;29]^. **b** Decrease of the HR-MDS mice survival compared to its single transgenic mice founders, _MRP8_NRASD12 and _tet_hBCL-2 transgenic mice; **c** Increased level of Lin-Sca1+-cKit+ (LSK) cells in the BM; **d** Increased apoptosis in the liver and spleen seen by whole body SPECT using ^99^Tc-Annexin (liver and spleen region located above the kidneys); **e** The disease can be transplanted in normal FVB/N irradiated syngeneic mice with either the Sca1+ cells of the spleen or BM of the HR-MDS mice resulting in 15 % blast infiltration in the BM blasts as in HR-MDS mice^[24-26;29]^

These mice mimic human high-risk MDS (HR-MDS) or AML post MDS depending on the promoter driving the transgene (mutant NRASD12 or hBCL-2) expression [32, 35]. BCL2 expression is required to drive the phenotype as inhibition of expression in conditional mice [32] or inhibition by BH3 mimetic inhibitors [29] reverts the phenotype. These models thus highlight the concept of non-oncogene addiction [36] where cells bearing the NRASD12 mutation require the BCL2 expression for survival and expansion. We have previously shown in these mice a concomitant increase in reactive oxygen species (ROS) with disease progression with a stepwise increase in the frequency of DNA damage leading to an increased frequency of error-prone repair of double-strand breaks (DSB) by nonhomologous end-joining [35]. The observed DNA damage and error-prone repair was decreased or reversed in vivo by *N*-acetyl cysteine antioxidant treatment [35], stressing the relevance of these mice models to relate genotype/phenotype to translational research. These models have already allowed to identify novel biomarkers of the disease in MDS patients such as the RAS:BCL-2 complex that links its localization at the plasma membrane or the mitochondria with the apoptosis features [30] and activates signaling protein profiles correlated with disease and progression to AML [29]. Both diseases are transplantable using BM or spleen cells from the diseased mice, and both transgenes are expressed at the stem cell level (Sca1+ compartment) [32]. Sca1− spleen cells had a much longer latency period before developing disease (Fig. 1e).

In this study, we found that the majority of the MDS-dysregulated pathways that were unique to the MDS mice and not the founder mice were close to those reported in human MDS patients, underscoring the relevance of these two mice models as preclinical models.

## Results and discussion

### Gene expression profile of the HR-MDS model

Establishment and characteristics of the HR-MDS model are summarized in Fig. 1a–e. It is obtained by crossing two single transgenic mice, expressing two human genes known to be implicated in MDS, mutated NRASD12 and BCL2 (Fig. 1a). The clinical and biological features of these transgenic mice have already been described [29, 30, 32–35], and the co-expression of the two transgenes is required to establish the HR-MDS disease and impact on survival, stressing the non-oncogenic addictive effect of the BCL2 expression (Fig. 1b). The main characteristics are pancytopenia, dysplasia, small percentage of blasts in the spleen and bone marrow (BM) (Fig. 1a, c), and increased apoptosis in the liver and spleen tissues by TUNEL [29] or by whole body SPECT using ^99^Tc-Annexin [29] (Fig. 1d). The model has been validated to mimic the clinical (survival, response to treatment) and biological features of HR-MDS in patients [29, 30, 32–35]. A novel biomarker, the NRASD12 and BCL2 plasma membrane complex, correlates with apoptosis in spleen and BM cells in the HR-MDS mice [32] and with apoptosis and low blast counts in MDS patients [30] underscoring the relevance of this model to study human MDS.

**Fig. 2:**
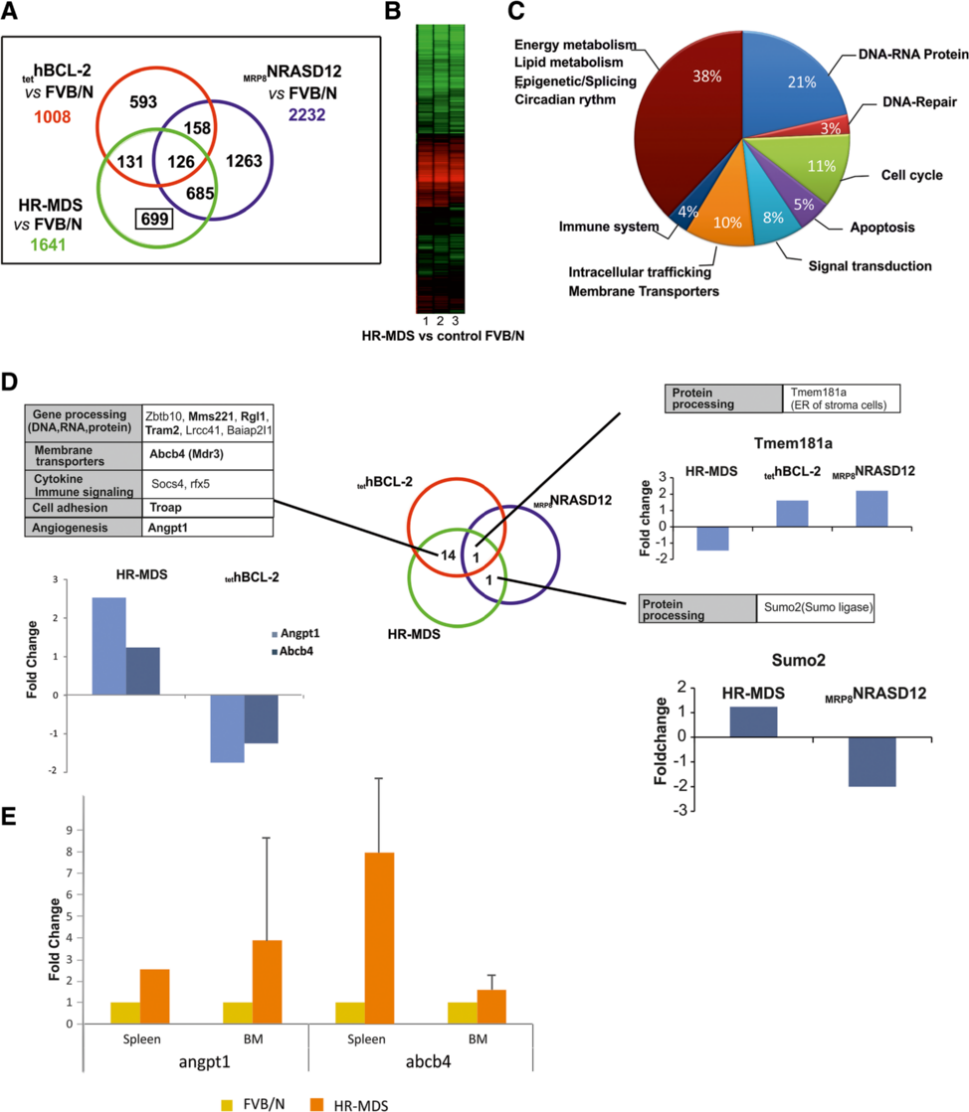
**a** Venn diagrams of co-expressed and uniquely dysregulated genes compared to control FVB/N mice in the HR-MDS, _MRP8_NRASD12, and _tet_hBCL-2 transgenic mice; **b** Heat map of the down- and upregulated genes performed on the Sca1+ cells of HR-MDS (*n* = 3) compared to control FVB/N (*n* = 3) mice; **c** Distribution in % of the significantly dysregulated genes in the KEGG David annotation pathways; **d** Analysis of the differentially expressed genes (i.e., upregulated in the HR-MDS transgenic mice and downregulated in the founder mice and vice versa). Genes in *regular font* are expressed at lower levels and genes in *bold font* are expressed in higher levels. Examples of microarray data are shown for each intersection; **e** Validation of microarray data. qRT-PCR fold change of angpt1 and abcb4 gene expression in the BM and spleen cells of a different set of HR-MDS mice

Thus, to further exploit and complete the description of this HR-MDS model, we performed gene expression profile (GEP) analysis. GEP analysis was performed on RNA extracted from Sca1+ cells from HR-MDS mice, the founder mice (_tet_hBCL2 and _MRP8_NRASD12) and normal FVB/N mice (Fig. 2a–c). Sca1+ spleen cells were chosen as we have shown that purified Sca1+ cells (from either the spleen or the BM of HR-MDS mice) (Fig. 1e) [32] can initiate the disease when transplanted in lethally irradiated syngenic FVB/N mice, and the spleen of these mice yields more cells than the bone marrow, a prerequisite for both RNA quality and quantity for the subsequent analyses. GEP results were however validated on both spleen and bone marrow samples (Figs. 2e and 4). Compared to normal FVB/N mice, 1641 genes were significantly dysregulated in HR-MDS mice, 1008 in the founder _tet_hBCL2 mice and 2232 in the _MRP8_NRASD12 mice. Fifty-six percent of these dysregulated genes were common and with similar levels of expression between the HR-MDS mice and its founders (_MRP8_NRASD12 and _tet_hBCL2) and were not further analyzed in this study (Fig. 2a and Additional file 1: Table S1). Gene set enrichment analysis (GSEA) was performed with the 1641 genes significantly dysregulated in HR-MDS mice compared to normal FVB/N mice, highlighting enrichment in cell metabolism (energy; lipid metabolism), cellular processes (DNA repair, cell cycle), angiogenesis, signal transduction, and immune system (Table 1 and Additional file 2: Figure S1A).

**Table 1:** Summary of the top pathways highlighted by GSEA on the total differentially expressed genes

HR-MDS versus FVB/N	AML post MDS versus FVB/N
*Adipogenesis*	*Adipogenesis*
*Androgen response*	**Allograft rejection**
**Angiogenesis**	*Androgen response*
*Bile acid metabolism*	Apical junction
*Coagulation*	*Bile acid metabolism*
***Complement***	**Cholesterol homeostasis**
*E2F targets*	*Coagulation*
Estrogen response late	***Complement***
**Fatty acid metabolism**	*E2F targets*
***G2M checkpoint***	epitheliall mesenchymal transition
***Glycolysis***	***G2M checkpoint***
Kras signaling	***Glycolysis***
***Mitotic spindle***	Hedgehog-signaling
mTORC1 signaling	***Mitotic spindle***
Myc targets	***Oxidative phosphorylation***
***Oxidative phosphorylation***	Pancreas beta cells
UV response-dn	Spermatogenesis
*Xenobiotic metabolism*	*Xenobiotic metabolism*

Italicized data indicate pathways in common between the two models; Bold data indicate similar pathways found in the « unique » genes

To select differentially expressed pathways implicated in the initiation or maintenance of the disease, we focused our study on a Database for Annotation, Visualization and Integrated Discovery (DAVID) GEP analysis performed on the 699 dysregulated genes that were unique to the HR-MDS mice (Fig. 2a and Additional file 1: Table S2) and the few dysregulated genes (*n* = 16) that were differentially expressed (i.e., upregulated in the HR-MDS and downregulated in the founder mice and vice versa) (Fig. 2d and Additional file 1: Table S3). Kyoto Encyclopedia of Genes and Genomes (KEGG) pathway analysis was performed using DAVID. The results are detailed in the Additional file 1: Tables S4–S14. The most significantly dysregulated genes were distributed into eight pathways (Fig. 2c). These dysregulated pathways included *gene processing* (namely DNA-RNA-protein processing) (Additional file 1: Table S4), *cellular processes* (DNA repair, cell cycle, survival/apoptosis) (Additional file 1: Tables S5–S7), *signal transduction* (Additional file 1: Tables S8 and S9), *intracellular trafficking* and *membrane transporters* (Additional file 1: Table S10), and *immune system* (Additional file 1: Table S11). Finally, around 38 % of dysregulated pathways concerned various pathways but with less genes involved per pathway including *energy and lipid metabolisms* (Additional file 1: Table S12), and *epigenetic/splicing* pathways (Additional file 1: Table S13)

**Fig. 3:**
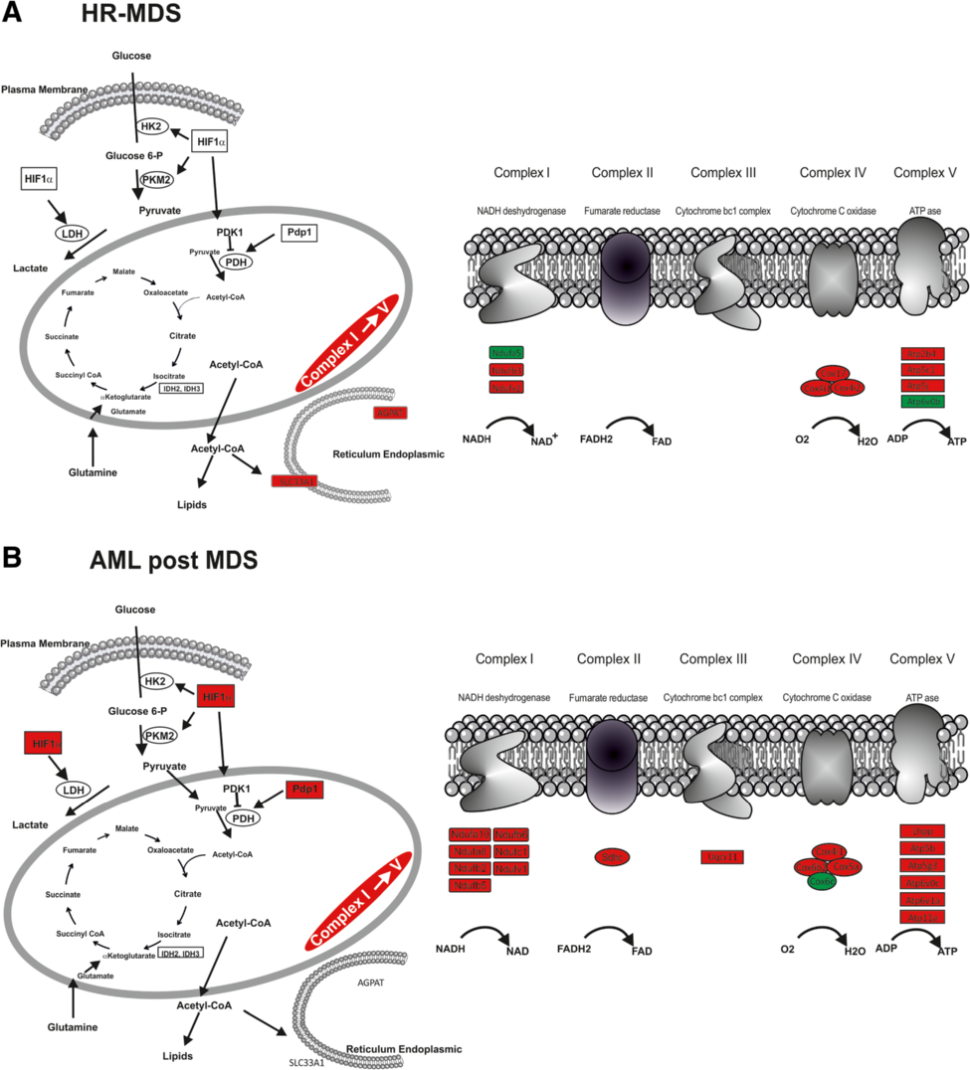
Schematic representation of dysregulated energy metabolism pathways. Dysregulated pathways are noted in *red*, if upregulated, and in *green*, if downregulated. **a** HR-MDS; **b** AML post MDS

**Fig. 4:**
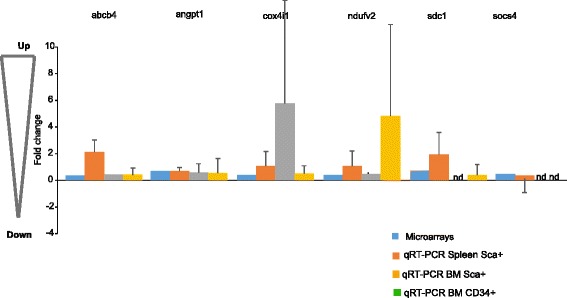
qRT-PCR validation of the GEP microarray data (*blue*) in Sca1+ cells purified from BM or spleen of another set of HR-MDS mice (*two different hues of orange*), and CD34+ cells purified from MDS patients (*green*) compared to FBV/N or CD34 BM cells, respectively. Data expressed as fold change of expression of several genes of three pathways shown (angpt1 (signal transduction); cox4i1 and ndufv2 (oxidative metabolism); sdc1 (DNA processing)). qRT-PCR fold changes shown here are represented as the average of three experiments

Of the ten most significantly upregulated pathways (Table 2), the pathway ranking first concerned genes of the PSM family of the proteasome, namely genes coding for different components of 26S. Increase in proteasome activity has been reported in MDS patients, and various studies have shown the potential benefit of combining an inhibitor of proteasome, bortezomid, with conventional MDS therapy [37, 38]. Equally significantly upregulated were genes coding for cell metabolism (energy and lipids) and the cell cycle/checkpoints/DNA repair. Genes coding for components of the major complexes of the mitochondrial electron transport chain were also significantly upregulated (Fig. 3a). These included genes of complex I: NADH deshydrogenase, complex IV: cytochrome c oxidase, and complex V: ATPase (confirmed by quantitative reverse transcription-PCR (qRT-PCR) in the BM and spleen cells of HR-MDS mice Fig. 4). Oxidative phosphorylation is the metabolic pathway in which mitochondria produce ATP required by proliferating cells. Oxidative metabolism also produces reactive oxygen species (ROS) such as superoxide and hydrogen peroxide, leading to propagation of free radicals, enhancement of antioxidant pathways but also DNA damage. Genes of the ROS/antioxidant pathways (such as *Txn1*, *Gpx1*, *Cebpα*) were not significantly dysregulated in the HR-MDS mice compared to the founder mice (Additional file 1: Table S14), whereas cell cycle checkpoints, DNA damage/repair genes were significantly upregulated (i.e., *mad2l1*, *cdc25a*, *mdm2*, *chek2*, *rad54b*, *brca1*, *fancm* (Table 2, Additional file 1: Tables S5 and S7). These pathways and genes have also been shown altered in some GEP studies of MDS patients [12, 16]. Amongst the lipid metabolism upregulated genes figured both those of ether lipid metabolism and glycosphingolipid biosynthesis. Though increase of acylglycerophospholipids and ether lipid metabolism have been reported in cancers, (confirmed by qRT-PCR in the BM and spleen cells of HR-MDS mice Fig. 4) with loss of tumorigenicity when efficiently targeted [39], little is known regarding MDS patients.

**Table 2:** Top regulated pathways in the list of upregulated genes in HR-MDS mice

Functional pathway KEGG database	*n*	Up *P* value	Upregulated genes^a^
*Genetic information processing* Proteasome	7	4.3E-04	**PSMD14, PSMD12, PSMC4, PSMA5, PSMD2, POMP, PSME4**
			*PSMD14*, *PSMD12*, *PSMC4*, *PSMA5*, *PSMD2*, *PSME4* : *PSM family (proteasome 26S subunit)*
			*POMP: proteasome maturation protein*
*Energy Metabolism* Oxidative phosphorylation	10	2.0E-03	**NDUFB3, NDUFV2, ATP5C1, COX4I2, COX4I1, VDAC2, VDAC3, ATP5J, SLC25A4, PPID**
			*NDUFB3*, *NDUFV2: NADH Ubiquinone oxido reductase*
			*ATP5C1*, *ATP5J: one of the subunits of mitochondria APTase*
			*VDAC2*, *VDAC3: voltage dependent anion channel.*
			*COX4I2*, *COX4I1cytochrome c oxidase subunit 4*
			*SLC25A4: SOLUTE CARRIER FAMILY 25 mitochondrial ADP/ATP translocator*
			*PPID: peptidylprolyl isomerase D*
*Lipid metabolism* Ether lipid metabolism	4	3.7E-02	**PAFAH1B3, AGPAT4, PPAP2A, CHPT1**
			*PAFAH1B3: platelet-activating factor acetylhydrolase 1b*, *catalytic subunit 3*
			*AGPAT4: 1-acylglycerol-3-phosphate O-acyltransferase 4*
			*PPAP2A: Phosphatidic Acid Phosphatase 2a*
			*CHPT1: choline phosphor transferase 1*
*Lipid metabolism* Glycosphingolipid biosynthesis	3	3.9E-02	**SLC33A1, HEXB, GLB1**
			*SLC33A1: solute carrier family 33 (acetyl-CoA transporter)*
			*HEXB: hexosaminidase B (beta polypeptide)*
			*GLB1 : Galactosidase*, *beta 1*
*Cellular processes* Cell cycle	7	5.4E-02	**MAD2L1, CCNH, SKP2, MDM2, CHEK2, CDC25A, BUB3**
			*MAD2L1*, *mitotic spindle assembly checkpoint (interacts with BUB1)*
			*CCNH: cyclin H; SKP2 : S-phase kinase-associated protein 2*
			*MDM2: mouse double minute 2 homolog (E3 ubiquitin-protein ligase); CHEK2: checkpoint*
			*BUB3: budding uninhibited by benzimidazole (checkpoint protein )*

^a^Gene card nomenclature

A survival/apoptosis pathway showed a significant downregulation of genes coding for inhibitors of apoptosis (*naip*, *tnfaip3*, and *madk9*) in line with the apoptosis observed in this HR-MDS model [29, 30, 32]. Downregulation of genes of the PI3K family (class II) and *Pten* was also significantly downregulated (Table 3). While *Pten* is a well-known tumor suppressor, few reports link it with MDS [40, 41]. Class II PI3K proteins are involved in the translocation of proteins to the cell membrane and have been shown instrumental in *Shh* signaling, a pathway implicated in the relation of stem cell with its environment [42]. Moreover, two other downregulated pathways included genes coding for *socs4*, a member of the suppressor of cytokine signaling family (confirmed by qRT-PCR in the BM and spleen cells of HR-MDS mice Fig. 4), and regulators of the immune response such as CD19 and CD40, also reported to be decreased in MDS [20, 22, 43]

**Table 3:** Top regulated pathways in the list of downregulated genes in HR-MDS mice

Functional pathway KEGG database	*n*	Down *P* value	Downregulated genes^a^
*Cellular processes* Inhibition of Apoptosis	5	1.2E-2	**NAIP6, NAIP5, NLRC4, MAPK9, TNFAIP3**
			*NAIP6; NAIP5: NLR family*, *apoptosis inhibitory protein 6; NLRC4: NLR family CARD domain-containing protein 4; MAPK9: Mitogen activated kinase 9; TNFAIP3: Tumor necrosis factor*, *alpha-induced protein 3*
*Environmental adaptation* Circadian rhythm	3	1.4E-2	**NR1D1, PER1, CLOCK**
			*NR1D1: nuclear receptor subfamily 1*, *group D*, *member 1*
			*PER1: period circadian clock 1*
			*CLOCK: circadian locomotor output cycles kaput*
*Signal transduction* Phosphatidylinositol signaling system	5	2.2E-2	**PIK3C2A, PIK3C2B, PIP4K2A, PTEN, ITPR1**
			*PIK3C2A: phosphatidylinositol-4-phosphate 3-kinase*
			*PIP4K2A: phosphatidylinositol-5-phosphate 4-kinase*, *type II*,
			*PTEN: phosphatase and tensin homolog*
			*ITPR1: inositol 1*,*4*,*5-trisphosphate receptor type 1*
*Human diseases* Type II diabetes mellitus	4	3.2E-2	**IRS2, MAPK9, CACNA1E, SOCS4**
			*IRS2: insulin receptor substrate 2; MAPK9: mitogen activated kinase 9*
			*CACNA1E: calcium channel*, *voltage-dependent*, *R type*, *alpha 1E subunit*
			*SOCS4: suppressor of cytokine signaling 4*
*Human diseases* Primary immunodeficiency	3	9.3E-2	**CD19, RFX5, CD40**
			CD19: antigen receptor of B lymphocytes; *RFX5: regulatory factor X*; CD40: TNF receptor superfamily member 5

When we separately studied the 16 genes that were differentially expressed between the HR-MDS mice and its founder single transgenic mice (i.e., upregulated in the HR-MDS and downregulated in the founders or vice versa), we found that the majority (14 out of 16) of these differentially regulated genes were between the HR-MDS and the founder _tet_hBCL-2 mice (Fig. 2d and Additional file 1: Table S3). The most significantly differentially dysregulated gene was angiopoietin, *Angpt1*, upregulated in the HR-MDS mice (confirmed by qRT-PCR in the BM and spleen cells of HR-MDS mice and CD34+ from MDS patients Figs. 2e and 4). Angiogenesis in MDS has been described, and some of the efficacy of Lenalidomide is related to its anti-angiogenic effect [44, 45]. The other dysregulated genes were linked to *gene processing* with transcription regulation genes such as *Zbtb10* (a Zinc finger/POZ domain gene), the *Mms22*l gene (a stabilizer of the NFKB-like enhancer), the *Rgl1* gene (the Ral guanine nucleotide dissociation stimulator involved in Ras and Ral signaling [46]), the *Lrrc41* gene coding for a substrate of RhoBTB-dependent *cullin* 3 ubiquitin ligase complexes [47], and the *Baiap2l1* gene that modulates *MDM2*-mediated p53 low-level ubiquitination. This latter gene is dysregulated in myelofibrosis and a fusion partner of *Fgfr3* in AML [48] *membrane transporters* with an increased expression of the P-glycoprotein gene (*Abcb4*-*Mdr3*) in the HR-MDS mice, gene linked to drug resistance in MDS [49] (confirmed by qRT-PCR in the BM and spleen cells of HR-MDS mice Fig. 4), *cytokine signaling* (*Socs4*, and *Rfx5*, a regulatory factor for major histocompatibility complex, altered in primary MHCI deficiency [50]) and *cell adhesion* with the *Troap* gene coding for the trophin-associated protein involved in cell adhesion complexes [51]. Only one gene, *Sumo2* (coding for a SUMO ligase) was significantly differentially expressed (upregulated) between the HR-MDS mice and its founder _MRP8_NRASD12 mice; SUMOylation is a major post translation modification of key proteins involved in cell control and carcinogenesis [52]. Finally, only one gene, *Tmem181a*, was differentially expressed between HR-MDS and its two founders, _MRP8_NRASD12 and _tet_hBCL-2 mice (upregulated in the founders and downregulated in HR-MDS mice). The function of Tmem181a is still not fully defined; it is a transmembrane protein with a conserved domain of Wnt binding factor required for Wnt secretion MIG-14 [53]. Abnormalities in the microenvironment and Wnt signaling may well relate to our current knowledge of MDS disease initiation and/or maintenance [54].

### Gene expression profile of the AML post MDS model

**Fig. 5:**
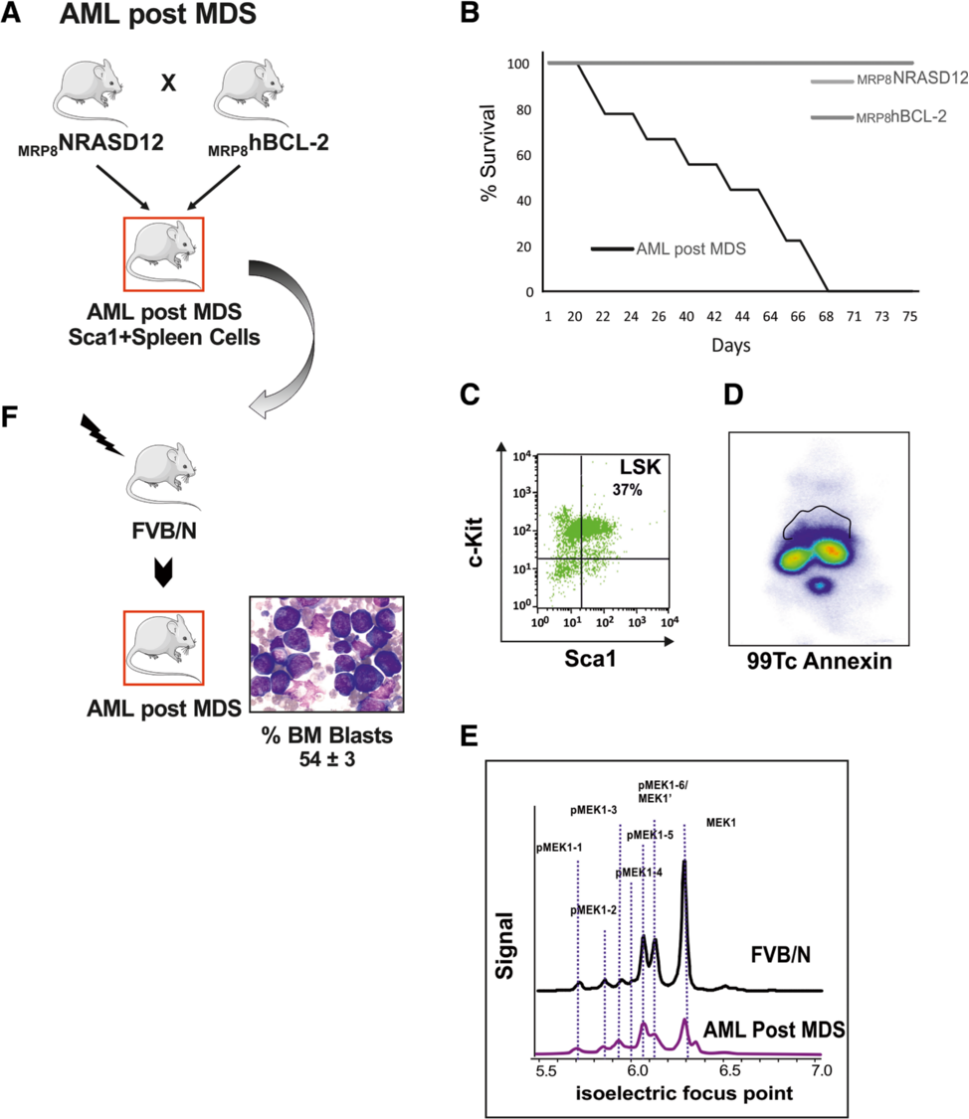
**a** Schematic representation of the AML post MDS mouse model. The characteristics of these models have previously been published ^[24-26;29]^; **b** Decrease of the survival of AML post MDS mice compared to the survival of the funders, _MRP8_NRASD12 and _MRP8_hBCL-2; **c** Increased level of Lin-Sca1+-cKit+ (LSK) cells in the BM; **d** Decreased level of apoptosis in the liver by whole body SPECT using ^99^Tc-Annexin (liver and spleen region located above the kidneys); **e** Validation of microarray data: Decreased levels of MEK isoforms in the AML post MDS spleen cells compared to FVB/N control mice by the nanofluidic proteomic analysis; **f** The disease can be transplanted in normal FVB/N irradiated syngeneic mice with either the Sca1+ cells of the spleen or BM of the AML post MDS mice resulting in 54 % blast infiltration in the BM blasts as in AML post MDS mice.^[24-26;29]^

The same GEP and functional annotation studies were performed in the AML post MDS model. The characteristics of the AML post MDS model are summarized in Fig. 5. The only difference in the establishment of the AML post MDS mice compared to that of the HR-MDS mice is the founder BCL2 mice where the promoter driving the expression of the BCL2 transgene is MRP8 (Fig. 5a). The AML post MDS mice have been described with the HR-MDS mice [29, 30, 32–35]. The co-expression of the two transgenes is required to establish the HR-MDS disease and impacts on survival (Fig. 5b). The AML post MDS mice present features of AML with major infiltration of blasts in the BM (Fig. 5a) and spleen, a significant increase of immature cells Lin^−^Sca1+ckit+ (LSK) cells in the BM (Fig. 5c) and absence of apoptosis by Tunel assay [29] and 99Tc-Annexin SPECT imaging (Fig. 5d). Persistence of dysplasia in myeloid cells confers the title of AML post MDS to the disease [25, 32]. We have previously shown in these mice an increase in reactive oxygen species (ROS) and the frequency of DNA damage [35]. Contrary to the HR-MDS mice, but in line with the leukemic features of these mice, the RAS:BCL2 complex, is here found at the mitochondria membrane, favoring BCL2 pathways and increased survival of blast cells [32]. The mitochondrial localization of the RAS:BCL2 complex was also noted in MDS patients with high blast count and low levels of apoptosis [30]. As for the HR-MDS model, we have shown that injection of Sca1+ spleen cells from AML post MDS mice transfers the disease in syngeneic mice (Fig. 5f) [32].

**Fig. 6:**
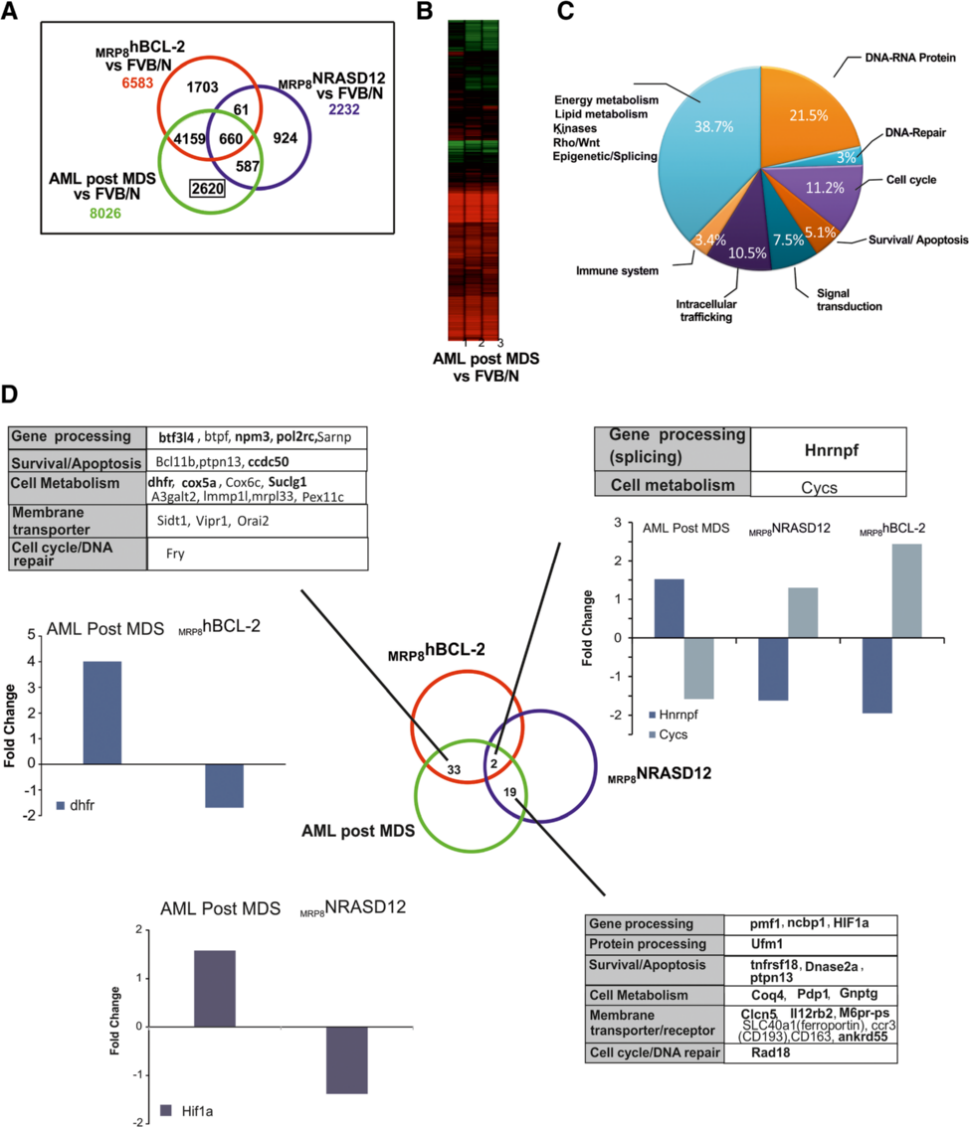
**a** Venn diagrams of co-expressed and uniquely dysregulated genes compared to control FVB/N mice in the AML post MDS, _MRP8_NRASD12 and _MRP8_hBCL-2 transgenic mice; **b** Heat Map of GEP performed on the Sca1+ cells of AML post MDS mice (*n* = 3) compared to control FVB/N mice (*n* = 3); **c** Distribution in % of the significantly dysregulated genes in the KEGG DAVID annotation pathways; **d** Analysis of the differentially expressed (i.e., upregulated in the AML post MDS transgenic mice and downregulated in the founder mice and vice versa). Genes in *regular font* are expressed at lower levels and genes in *bold font* are expressed in higher levels. Examples of microarray data are shown for each intersection

The GEP of these mice shows a total of 8026 dysregulated genes compared to normal FVB/N mice (Fig. 6a, b), as most (67 %, *n* = 5352) of these dysregulated genes were common and with similar levels of expression between the AML post MDS mice and the founders (the _MRP8_NRASD12 or the _MRP8_hBCL2 single transgenic mice) (Additional file 1: Table S15). Gene Set Enrichment Analysis (GSEA) was performed with the 8026 genes significantly dysregulated in AML post MDS mice compared with normal FVB/N mice, highlighting enrichment in *cellular processes* (cell cycle/DNA damage-repair), cell metabolism (energy; lipid metabolism), and immune system (Table 1 and Additional file 2: Figure S1B).

As for the HR-MDS model, to select differentially expressed pathways implicated in the initiation or maintenance of the disease, we focused our study on Database for Annotation, Visualization and Integrated Discovery (DAVID) GEP analysis, on the 2620 dysregulated genes that were unique to the AML post MDS mice (Additional file 1: Table S16) and the 54 dysregulated genes that were differentially expressed (i.e., upregulated in the AML post MDS and downregulated in the founder mice and vice versa) (Fig. 6d and Additional file 1: Table S17).

We first analyzed the 2620 genes unique to the AML post MDS mice (Additional file 1: Table S16). DAVID and KEGG functional annotation distributed the AML post MDS dysregulated genes in functions similar to those noted for the HR-MDS model, underscoring the malignant background of both models (Fig. 6c). Like the HR-MDS mouse, the majority of the genes were distributed in pathways implicated in *gene processing* (DNA-RNA-protein) (Additional file 1: Table S18), DNA-repair (Additional file 1: Tables S19), *cellular processes* (cell cycle (Additional file 1: Tables S20), survival/apoptosis (Additional file 1: Table S21), signal transduction (Additional file 1: Tables S22, S23), intracellular trafficking, membrane transporters (Additional file 1: Table S24)), and *genes of the immune system* (Additional file 1: Tables S25, S26). Other pathways included *energy and lipid metabolism* (Tables 4 and 5; Additional file 1: Table S28), *kinases* (Additional file 1: Table S23), *Rho/Wnt* pathway (Table 6 and Additional file 1: Table S23C), and genes of *splicing* and *epigenetic* enzymes (Additional file 1: Tables S18, S27).

**Table 4:** Top regulated pathways in the list of upregulated genes in AML post MDS mice

Functional pathway KEGG database	*n*	Up *P* Value	Upregulated genes^a^
*Cell metabolism* Oxidative phosphorylation	18	1.0E-03	**NDUFB5, NDUFB6, NDUFB8, ATP5B, COX4I1, NDUFC1, COX5A, LHPP, NDUFA10, ATP5G3, PPA2, NDUFB2, ATP6V0C, ATP6V1A, UQCR11, NDUFV1, SDHC, COX6B2**
			NDUF family: NADH dehydrogenase (ubiquinone) Fe-S family
			SDHC: succinate dehydrogenase complex, subunit C
			LHPP: phospholysine phosphohistidine inorganic pyrophosphate phosphatase
*Nucleotide metabolism* Pyrimidine metabolism	14	2.9E-03	**POLR2G, NUDT2, DTYMK, POLE, AK3, POLA1, POLR2C, NME4, TYMP, POLE3, NME1, POLD1, ENTPD1, DUT**
			NUDT2: nucleoside diphosphate, AK3: adenylate kinase
			TYMP: thymidine phosphorylase, NME: nucleoside diphosphate kinase
			POLE, POLR2C, POLE3, PLD1: polymerases, DUT: deoxyuridine triphosphate
*Immune system* Toll-like receptor signaling	14	3.8E-03	**MAP2K3, MAP2K4, TLR4, FADD, CXCL11, TLR7, CCL4, TLR8, CD80, IFNA7, MAPK13, IFNA5, TICAM2, PIK3R2**
			MAP2K3, MAP2K4: MEK proteins, TICAM2: TIR domain-containing adapter molecule 2
			PIK3R2: Phosphatidylinositol 3-kinase regulatory subunit beta
*Cellular processes* Cell cycle	16	5.8E-03	**CDC6, E2F3, CCNH, SKP2, YWHAB, ESPL1, CDC20, CHEK1, CHEK2, CDC25C, MCM4, 2CDK2, CCNE2, CDC45, MAD2L1, CDKN2C**
			CCNH: cyclin H; SKP2: S-phase kinase-associated protein 2; YWHAB: 14-3-3 protein beta/alpha
			ESPL1:separin, CCNE2: cyclin E2, MAD2L1: MAD2 mitotic arrest deficient-like 1
			CDKN2: cyclin-dependent kinase inhibitor 2A (p16)
*Signaling molecules* Cytokine-cytokine receptor	25	5.9E-03	**IL1R1, ACVRL1, PDGFB, IL13, CXCL11, CCL4, TNFRSF4, IL10, IL12RB2, CCL22, IFNA7, IFNA5, TNFRSF18, IFNGR1, EPO, IL4, IL23R, IL21, CCL17, TNFRSF9, INHBA, CCR8, TNFSF11, CXCL13, CCR2**
			IL1R1, IL12RB, I L23R,IL21: interleukines/interleukine receptor
			CCL4, CCL22, CCL17, CXCL11, CXCL13 CCR2, CCR8: chemokine
			TNFRSF4, TNFRSF18, TNFRSF9, TNFSF11. Tumor necrosis receptor
			IFNA7, IFNA5, INFGR1, INHBA: interferon signaling
			EPO: erythropoietin
*Signal transduction* JAK-STAT signaling pathway	15	5.0E-02	**IL4, IL23R, SOCS2, IL13, IL21, IL10, IL12RB2, SPRY1, IFNA7, IFNA5, SPRED2, IFNGR1, IL13RA2, EPO, PIK3R2**
			IL4, IL23R, IL13, IL21, IL10, IL12RB2, IL13RA2: interleukines/interleukine receptor
			IFNA7, IFNA5, IFNGR1: interferon signaling
			EPO: erythropoietin
			PIK3R2: phosphoinositide-3-kinase, regulatory subunit 2
*Genetic information processing.* Replication and repair	5	6.4E-02	**BLM, RAD51L1, POLD1, RAD54B, RAD51**
			BLM: bloom syndrome protein, POLD1: polymerase delta
			RAD51L1, RAD54B, RAD51: recombinases family
*Genetic information processing*	11	6.8E-02	**HIST1H2AC, C4A, H2AFV, CD80, HIST2H2BE, HIST2H2AC, HIST1H2BH, HIST1H2AI, SNRPB, H2AFY, FCGR1, IL10**
			HIST1H2AC,H2AFV,HIST2H2BE,HIST2H2AC,HIST1H2BH, HIST1H2AI, H2AFY: histone proteins
			C4A: complement factor 4; CD80, costimulatory factor, SNRPB: small nuclear ribonucleoprotein-associated proteins B, FCGR1: IgG Fc receptor

^a^Gene card nomenclature

**Table 5:** Top regulated pathways in the list of downregulated genes in AML post MDS mice

Functional pathway KEGG database	n	Down *P* value	Downregulated genes^a^
*Cellular processes* Inhibition of Apoptosis	5	1.2E-2	**NAIP6, NAIP5, NLRC4, MAPK9, TNFAIP3**
			*NAIP6; NAIP5: NLR family*, *apoptosis inhibitory protein 6; NLRC4: NLR family CARD domain-containing protein 4; MAPK9: mitogen activated kinase 9; TNFAIP3: tumor necrosis factor*, *alpha-induced protein 3*
*Environmental adaptation* Circadian rhythm	3	1.4E-2	**NR1D1, PER1, CLOCK**
			*NR1D1: nuclear receptor subfamily 1*, *group D*, *member 1*
			*PER1: period circadian clock 1*
			*CLOCK: circadian locomotor output cycles kaput*
*Signal transduction* Phosphatidylinositol signaling system	5	2.2E-2	**PIK3C2A, PIK3C2B, PIP4K2A, PTEN, ITPR1**
			*PIK3C2A: phosphatidylinositol-4-phosphate 3-kinase*
			*PIP4K2A: phosphatidylinositol-5-phosphate 4-kinase*, *type II*,
			*PTEN: phosphatase and tensin homolog*
			*ITPR1: Inositol 1*,*4*,*5-trisphosphate receptor type 1*
*Human diseases* Type II diabetes mellitus	4	3.2E-2	**IRS2, MAPK9, CACNA1E, SOCS4**
			*IRS2: insulin receptor substrate 2; MAPK9: mitogen activated kinase 9*
			*CACNA1E: calcium channel*, *voltage-dependent*, *R type*, *alpha 1E subunit*
			*SOCS4: suppressor of cytokine signaling 4*
*Human diseases* Primary immunodeficiency	3	9.3E-2	**CD19, RFX5, CD40**
			CD19: antigen receptor of B lymphocytes; *RFX5: regulatory factor X* ; CD40: TNF receptor superfamily member 5

**Table 6:** Genes implicated in WNT signaling differentially dysregulated in the AML post MDS transgenic mice compared to control FVB/N mice

Gene symbol	Gene name	Regulation	Fold change	*P* value
Mesdc2	Mesoderm development candidate 2	Up	1.98	1.92E-02
Csnk2b	Casein kinase 2, beta polypeptide	Up	1.88	1.76E-02
Fzd7	Frizzled homolog 7	Up	1.75	2.74E-03
Ctnnb1	Catenin (cadherin associated protein), beta 1	Up	1.74	4.24E-04
Bambi	BMP and activin membrane-bound inhibitor, homolog (Xenopus laevis)	Up	1.73	1.56E-02
Dab2	Disabled homolog 2	Up	6.67	2.12E-02
Cd44	CD44 antigen	Up	1.68	5.96E-03
Wdr61	WD repeat domain 61	Up	1.64	7.57E-04
Sdc1	Syndecan 1	Up	3.11	5.94E-03
Hhex	Hematopoietically expressed homeobox	Down	1.98	2.46E-02
Macf1	Microtubule-actin crosslinking factor 1	Down	1.94	8.80E-03
Mark2	MAP/microtubule affinity-regulating kinase 2	Down	1.94	2.41E-02
Tnik	TRAF2 and NCK interacting kinase	Down	1.91	3.10E-03
Tle1	Transducin-like enhancer of split 1, homolog of Drosophila E(spl)	Down	1.88	1.51E-02
Dvl2	Dishevelled 2, dsh homolog (Drosophila)	Down	1.67	7.34E-03
Csnk1a1	Casein kinase 1, alpha 1	Down	1.65	1.27E-02
Rock1	Rho-associated coiled-coil containing protein kinase 1	Down	1.61	7.04E-03
Map3k7	Mitogen-activated protein kinase kinase kinase 7	Down	1.58	9.96E-04
Smad4	MAD homolog 4	Down	1.54	6.36E-03
Dapk3	Death-associated protein kinase 3	Down	1.52	1.06E-02
Tbl1xr1	Transducin (beta)-like 1X-linked receptor 1	Down	1.52	8.39E-04
Ppp3ca	Protein phosphatase 3, catalytic subunit, alpha isoform	Down	1.50	9.70E-03
Ppap2b	Phosphatidic acid phosphatase type 2B	Down	2.10	2.74E-02

KEGG analysis identified 13 significantly dysregulated pathways (Tables 4 and 5). The most upregulated pathway concerned *cell metabolism* (Table 4)*.* Indeed, in this model, all the components of the oxidative phosphorylation pathway were upregulated (Fig. 3b) (complex I: NADH dehydrogenase, complex II: fumarate reductase, complex III: cytochrome bc1 complex, complex IV: cytochrome c oxidase, and complex V: ATPase). Amongst the other upregulated cell metabolism pathways were those of *nucleotide/pyrimidine metabolism*. The upregulation of these pathways are in line with the up regulation of other *cellular processes* pathways such as: cell cycle, DNA replication and repair (*blm*, *rad18*, *rad51*, *rad54*), and multiple histone family genes, signaling molecules and signal transduction (cytokine and cytokine receptors including Epo, chemokines, TNFs, JAK-STAT signaling), and the *immune system* with Toll receptor and interferon pathways. All these pathways find their place in the active leukemia features found in this mouse model and have to some extent been reported either in MDS or AML patients [20, 22, 29, 30, 32–35, 43]. Interestingly, one study also highlighted the upregulation of histone genes coding for histones 2 and 1 in MDS patients [55].

Intriguingly, of the five most downregulated pathways (Table 5), three concern *signal transduction pathways*. The most significantly downregulated pathway was represented by numerous kinases of the MAPK (MEK) pathway (MAP2K1, MAPK2P2 MAKA4P2, MAP3K7). These MAPK genes also cluster in two functional annotation signal transduction pathways (*signal transduction and pathways in cancer*) with other enzymes or proteins acting downstream of environmental signaling such as *bmp*, *Tgfβ*, or *Wnt* signaling. We confirmed the decreased expression of one of these MAPK, MAP2K, at the protein level in another set of AML post MDS mice cells by nanofluidic proteomic immunoassay (Fig. 5e). The *Wnt pathway* (found in the annotation “*cancer pathways*”) is the most targeted pathway with 23 dysregulated genes (Tables 4 and 6). Fourteen of these genes were downregulated (such as *Dvl*, *Ppp3ca*, *Csnk1a1*, *Smad4*, *Rock1*) while two genes coding for inhibitors of Wnt signaling, *Icat* (*Ctnnbip1*) and Disabled (*Dab2*) were upregulated (the latter by nearly sevenfold). Other pathways pointed to a potential dysfunction of interaction with the environment, such the Rho signaling pathway (Additional file 1: Table S23C) and the CD44 gene (Table 6). The downregulation of the Wnt canonical pathway has already been underscored in several studies in MDS patients [14, 19, 20, 22, 24, 56, 57]. However, our analysis also highlights that genes of the noncanonical Wnt pathway (*Rock1*, *Can*, *Ppp3ca*, and *Rho/Rac* GEF) are also altered. These combined altered pathways underscore the importance of the regulation of genes of the microenvironment in this disease [58]. Finally, in this model, genes of the class II PI3K family and *Pten* were also downregulated as observed in the HR-MDS model (Tables 2, 4, and 5).

As in the HR-MDS GEP study, we analyzed the 54 genes that were differentially expressed between the AML post MDS model and its founders (i.e., upregulated in the AML post MDS and downregulated in the founders or vice versa) (Fig. 6d and Additional file 1: Table S15). These genes predominated in the functional pathway *gene processing* (DNA-RNA-protein) such as *pmf1*, *btf3l4*, *bptf*, *npm3*, *pol2rc*, *ncbp1*, *Ufm1*, *hnrnpf*, and *Hif1α*. Several of these genes are implicated in leukemogenesis such as the npm genes (*nmp1* and *nmp3*). *HIF1α* is a key partner of glycolysis (Fig. 3b) [59] and a target gene of *E2F3* [60]. Both *HIF1α* (Additional file 1: Table S17) and *E2F3* (Additional file 1: Table S16) genes are upregulated in the AML post MDS. MDS patients have been reported to express high levels of *E2F3* and *dhfr* [61] and Hif1α [62] underscoring the relevance of the mice model. Genes of *cellular processes* such as survival/apoptosis, cell cycle, and DNA repair were also differentially expressed. Genes of *cell metabolism* were also identified such as genes coding for succinate-CoaA ligase, cytochrome c oxidase (*Coxa5*, *Cox6*), *Coq4*, or dihydrofolate reductase (*dhfr*). Two genes (*cysc* and *hnrnpf*) were dysregulated between the AML post MDS mice and both founder. The *Hnrnpf* gene (heterogeneous nuclear ribonucleoprotein F) is involved in the regulation of alternative splicing [63] and has been found altered in myeloid malignancies [63] while the *cysc* gene codes for cytochrome C in the electron respiratory chain and is also a major mitochondrial initiator of the apoptosis intrinsic pathway (Fig. 3b). Finally, other dysregulated genes involved *membrane transporter genes* (such as iron (*Slc40a1*/ferroportin), calcium (*Orai2*), H+/Cl- (*clnc5*), small RNAs (*Sidt1*), ankyrine (*ankrd55*) or *membrane receptor genes* (vasopressine (*Vipr1*), scavenger (CD163), mannose phosphate (*M6pr-ps*), chemokine (*ccr3*), and IL12 (*IL12tb2*). Of note, the role of the iron transporter was reported in MDS [64].

### Validation of GEP

The GEP microarray data was validated on a different set of HR-MDS mice produced a year after the mRNA extraction for the GEP analysis on both BM and spleen Sca1+ purified cells. A similar dysregulation of gene expression was noted on three selected pathways, *angpt1* (signal transduction), *cox4i1* and *ndufv2* (oxidative metabolism), and *sdc1* (DNA processing). For all these genes, there was a concordance between (a) the array analysis and the RT-PCR study and (b) a concordance between the spleen and bone marrow gene expression validating the study performed on spleen cells (Fig. 4). We also confirmed the decreased expression of one of these MAPK, MAP2K, at the protein level in another set of AML post MDS mice cells by nanofluidic proteomic immunoassay (Fig. 5e).

The relevance of the GEP results was also validated in CD34+ BM cells of MDS patients (*n* = 3) on the same gene panel. For these pathways (signal transduction, oxidative metabolism, and DNA processing), the dysregulation was the same in the BM of HR-MDS mice and MDS patient CD34+ BM cells when compared to normal FVB/N control cells and normal CD34 BM cells (Fig. 4). This is the only discrepancy we noted when comparing the HR-MDS mice GEP data with gene expression data in MDS patient samples (whether from data of the literature or validation analysis performed in this study). However, the complete disruption noted in the MDS patient samples is in line with that reported in patients with solid tumors [65, 66] and strengthens their potential relevance to human MDS though how these pathways impact on the disease still needs to be elucidated as in solid tumors.

Thus, the GEP analysis of these mice, at the gene expression level, validates them as unique models of human MDS, at two different stages of the human MDS disease, high risk (HR-MDS), and at transformation (AML post MDS). Though published MDS mice models have provided insights to initiating mechanisms such as splicing abnormalities, chromosomal deletions, or abnormalities of the microenvironment [25, 67], most are characterized by myeloid proliferation, whether chronic or acute, with little reported analysis of apoptosis, and very few have achieved a translational benefit.

**Fig. 7:**
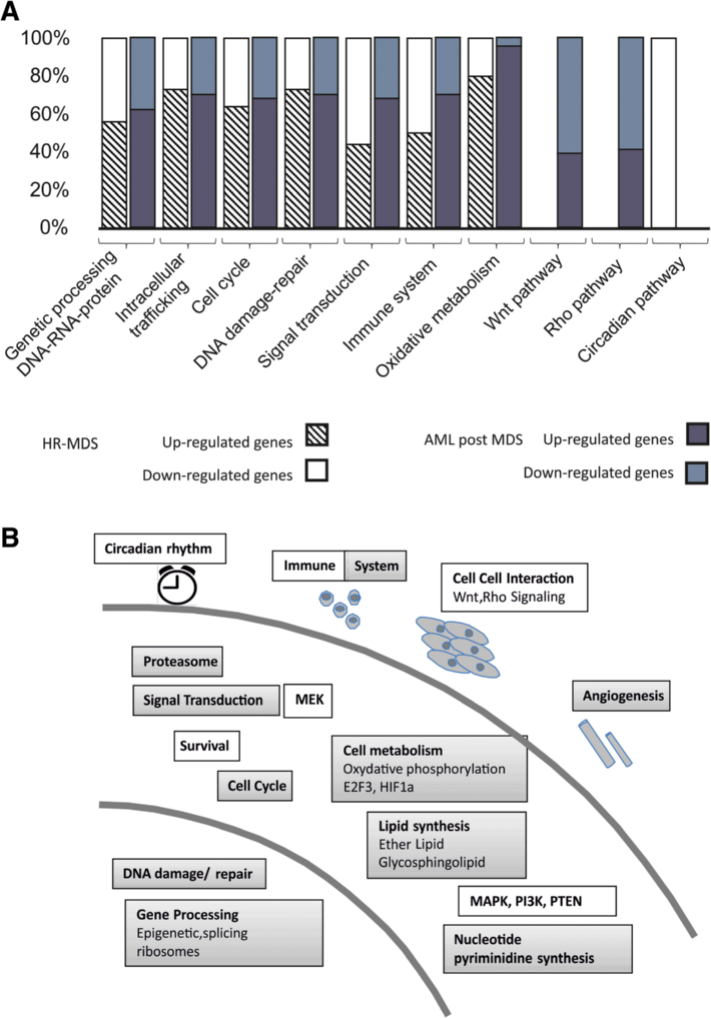
**a** Frequency in percent of the most significant pathways dysregulated (up- or downregulated) in the two MDS mice models; **b** Schematic summary of the dysregulated pathways noted in the two MDS mice models. Upregulated pathways (*gray boxes*), downregulated pathways (*white boxes*)

The majority of the identified pathways in the HR-MDS and AML post MDS mice identified by this GEP analysis are similar to those reported in similar annotation studies performed in MDS patients, when all analyses are combined [11–14, 16, 18–22, 24, 43, 45, 56–58, 64, 68–77]. Most of the dysregulated pathways were expected in a malignant context, such as pathways of gene processing, intracellular trafficking, cell cycle, DNA damage-repair, signal transduction, the immune system, and oxidative metabolism (Fig. 7a), and confirmed by GSEA analysis performed on all genes dysregulated in HR-MDS (Additional file 1: Table S29, Additional file 2: Figure S1A) or AML post MDS (Additional file 1: Table S30, Additional file 2: Figure S1B) vs FVB/N mice. The latter three annotation pathways are more frequently dysregulated in the AML post MDS mice. A more focused analysis on pathways now known to be implicated in MDS and AML [3, 18] also identified dysregulation of genes coding for epigenetic proteins (Additional file 1: Table S13 and S27), enzymes involved in methylation and acetylation processes (Additional file 1: Tables S4, S18 and Additional file 3: Figure S2D) as well as genes implicated in splicing and translation in both diseases (Additional file 1: Tables S4, S16, S18), confirming the importance of altered epigenetic, splicing, or ribosomal pathways in MDS diseases whether they are induced by mutations or altered gene expression. Thus, both mice models may be useful to further analyze and target these known pathways.

The GEP and annotation analysis equally identified novel dysregulated pathways, most of which have been identified in the solid tumor field but not as yet in AML or MDS. These pathways, detailed above, concern lipid and ether metabolism, the proteasome, MAPKinases, and more specifically, the noncanonical Wnt (Fig. 7b). The dysregulation of these pathways was confirmed by the differential gene expression (or nanofluidic proteomic immunoassay) seen in the BM and spleen cells of a different set of MDS mice and in the BM CD34+ cells of MDS patients when compared to their normal counterparts (Figs. 2e, 4). How these dysregulations occur and impact on the initiation or maintenance of MDS and in the networks of the other dysregulated pathways will require further studies.

## Conclusions

In conclusion, this study confirms that the HR-MDS and AML post MDS mice mimic two stages of the human MDS disease not only in their clinical and biological level but also in their gene expression level. This study further highlights novel less well studied pathways such as energy and lipid metabolism and noncanonical Wnt signaling which may concur with epigenetics and DNA damage to the genomic instability of these diseases. These MDS models should help unravel the underlying networks at the origin of these dysregulation and provide further biomarkers and targets for MDS disease.

## Methods

### Transgenic mice

The _MRP8_[NRASD12/hBCL-2] (AML post MDS) and _MRP8_NRASD12/_tet_hBCL-2 (HR-MDS) transgenic mice were generated from crosses of _MRP8_hBCL-2 or _MMTVLTRtTA/Teto_hBCL-2 (_tet_hBCL-2) with _MRP8_NRASD12 hemizygote mice in FVB/N, as previously described [33, 34]. For genotyping, DNA was extracted from the tails of 3-week-old mice using the KAPA Express Extraction Kit using manufacturer instructions. The PCR reaction was then performed with REDTaq® ReadyMixTM PCR Reaction Mix with MgCl2. Mice were classified according to the Bethesda classification [78] as HR-MDS or AML post MDS-like by cytological and pathology criteria. Mice were maintained in the barrier facilities of the Institut Universitaire d’Hématologie. Procedures involving animals and their care conformed with institutional guidelines that comply with national and international laws and policies and were authorized by the local ethical committee. Mice were sacrificed when they were moribund or upon veterinary advise. Committee on the Ethics of Animal Experiments Paris-Nord: C2EA-121 approved this project no. 2014IUH006

### Cell and tissue preparation, flow cytometry

The bone marrow (BM) was obtained by flushing long bones with Hank’s balanced salts solution followed by filtering through a nylon mesh. BM smears were prepared according to standard hematological techniques. BM smears were stained and examined by a cytologist (MEN) of Hôpital Saint-Louis. Percentage blasts were determined from the BM smears by counting 100–200 cells. Spleen cells from AML post MDS mice (day 36 post birth) and HR-MDS mice (day 80 post birth) were obtained by soft dilaceration of the spleen with the piston of a 5-ml syringe in a petri dish. Cells were washed in PBS, filtered through a 40-μm nylon mesh and then density centrifugation was conducted using Lymphoprep (Eurobio, France) to isolate mononuclear cells. Spleen or BM cells were labeled with anti-Sca1+ antibodies coupled with microbeads from Miltenyi and then sorted using an AutoMacs separator (Miltenyi Biotec Bergisch Gladbach, Germany). 5.10^6^ to 10^7^ Sca1+ sorted cells were used to extract total RNA using TRIzol (Invitrogen, CA, USA). Quantification and quality of the RNAs were assessed using a Nanodrop (Thermoscientific, USA).

### Secondary transplantation of bone marrow and spleen cells

Bone marrow cells, isolated from long bones, and spleen cells were harvested from 6- to 10-week-old AML post MDS mice as described above, pooled and divided (10^7^ nucleated cell aliquots per recipient) for i.v. injections into 12 irradiated FVB/N mice. Six- to 8-week-old FVB/N mice were prepared for transplantation by cesium irradiation totaling 10 Gy, divided into two doses 3 to 4 h apart. Successful transfer of the transgene-positive cells was confirmed by PCR. Spleen cells (10^5^, 10^6^, or 10^7^) from HR-MDS mice were injected i.v. in tail veins of immunocompromised RAG1-deficient mice, which are B cell and T cell deficient. [79]

### Nanofluidic proteomic immunoassay (NanoPro assay)

Splenocyte cells (10^6^) isolated from transgenic mice were lysed in radio immunoprecipitation assay buffer and subjected to a nanofluidic proteomic immunoassay (NIA) run on the NanoPro1000, as we have previously described [80] (ProteinSimple, Santa Clara, CA) mixing 0.1 mg/mL of lysate in a final volume of 15 μL loaded in a 384-well plate with five to eight gradient ampholyte mix. Antibodies were diluted in ProteinSimple antibody diluent: mitogen-activated protein kinase (MEK)1 (Upstate 07-641; Millipore, Billerica, MA) 1:100. Samples were run in triplicate.

### ^99^Tc-Annexin scintigraphy

Single-Photon Emission Computed Tomography (SPECT) was performed under pentobarbital anesthesia (4 mg/100 g body weight; Ceva Santé Animale, Libourne, France) in mice, after intravenous injection of ^99^Tc-Annexin. Images were acquired 10 min after injected dose of ^99^Tc-Annexin. Planar images were obtained 0 to 45 min (dynamic acquisition: 15 images, image duration: 60 s, static acquisitions of 10 min duration) after ^99^Tc-Annexin injection. In addition, mice which had previously undergone planar imaging underwent abdominal X/tomoscintigraphy acquisition: mod-list tomographic acquisition was performed during continuous rotation of the animal placed between two parallel collimators (360° rotation per minute, acquisition duration: 60 min from 1 h to 2 h after ^99^Tc-Annexin injection). All acquisitions were performed using a dedicated small animal IMAGER-S/CT system (Biospace Mesures, Paris, France) equipped with parallel low-energy high-resolution collimators (matrix 128 × 128, 15 % energy window centered on 140 KeV). ^99^Tc-Annexin uptake in hepato-splenic area was visually assessed.

### Affymetrix exon array hybridization

One microgram of total RNA extracted from Sca1+ spleen cells from the AML post MDS mice, HR-MDS mice, single-transgenic mice necessary to produce these AML post MDS and HR-MDS mice, (_MRP8_NRASD12, _MRP8_hBCL-2, _tet_hBCL-2) (called founder mice), and FBV/N control mice (*n* = 3 each) was labeled with Affymetrix reagents and hybridized to Affymetrix-GeneChip Mouse Exon 1.0 ST arrays. Affymetrix Expression Console Software was used to perform quality assessment.

For each of the 18 arrays (_MRP8_NRASD12/_tet_hBCL-2, _MRP8_[NRASD12/hBCL-2], _MRP8_NRASD12, _MRP8_hBCL-2, _tet_hBCL-2, and the control FVB/N mice), 100 ng of total RNA was first mixed with bacterial transcripts and the mixture was reverse transcribed into complementary DNA (cDNA). After synthesis of double-stranded cDNA, an in vitro transcription reaction was conducted overnight. Resulting amplified cRNA was reverse transcribed into sense DNA incorporating dUTP. This single-stranded DNA was treated with a combination of uracil DNA glycosylase and apurinic/apyrimidinic endonuclease 1. DNA fragments were biotin-labeled by terminal deoxynucleotidyl transferase. Targets were finally prepared according to Affymetrix recommendations for hybridization of exon arrays. Microarrays were hybridized, washed, and scanned using Affymetrix instruments. Total RNAs RIN values were between 8.3 and 9. Raw data are controlled with Expression console (Affymetrix).

### Microarray data and statistical analysis

Affymetrix exon array data analyses were performed using EASANA based on FAST DB annotations [81] (GenoSplice technology; http://www.genosplice.com/). Unpaired statistical analyses were performed using Student’s paired *t* test as previously described [82]. Affymetrix Expression Console Software was used to perform quality assessment. All array present a “pos_vs_neg_auc” value greater or equal than 0.82; All arrays present a “all_probeset_rle_mean” value lesser or equal than 0.35; All arrays present a “%” value greater or equal than 65 % and less than 6 % of difference between arrays from the same experimental condition.

Regarding the expression status/background noise analysis, only probes with a DABG *P* value ≤0.05 in at least half of the arrays from a given experimental condition were considered for further statistical analysis.

We kept a cutoff of *P* value ≤ 0.05 of increase or decrease of expression compared to normal samples for the analysis [83], Gene Set Enrichment Analysis (GSEA), and gene ontology using the Database for Annotation, Visualization and Integrated Discovery (DAVID) (http://https://david.abcc.ncifcrf.gov) were performed [84–87]. Though our analysis is focused on the unique genes dysregulated in the HR-MDS and the AML post MDS transgenic mice and sets of genes differentially expressed in HR-MDS or AML post MDS and founders, the data from all mice was analyzed as of potential relevance to the initiation of the disease (Additional file 1: Tables S1-28). This resulted in a selection of 2674 genes unique for AML post MDS and 715 for HR-MDS that were used for functional analysis. Microarray data from this study were included in the NCBI Gene Expression Omnibus no. GSE72934.

### HR-patient samples

BM aspirates from MDS patients were obtained in accordance with the Declaration of Helsinki. Participants provide their written informed consent to participate in Cancer research. The study presented here is part of a study approved by “Comité d’Evaluation de l’Ethique des Projets de Recherche Biomédicale” (CEERB) Paris Nord—IRB00006477—on 17 June 2013 (opinion number 13-027).

Patients had been diagnosed according to the World Health Organization (WHO) 2008 classification [88, 89]. CD34+ enriched cells were obtained by using anti-CD34+ antibodies coupled with microbeads from Miltenyi and then sorted using an AutoMacs separator (Miltenyi Biotec Bergisch Gladbach, Germany). CD34+ sorted cells were used to extract total RNA using TRIzol (Invitrogen, CA, USA).

### Real-time RT-PCR

Total RNA (1 μg) was reverse transcribed with SuperScript III reverse transcriptase (Invitrogen) using 100 ng of Random Hexamers. Quantitative real-time RT-PCR was performed in a test set of HR-MDS mice (*n* = 3) Sca1+ enriched BM and spleen cells and MDS patients (*n* = 3) CD34+ enriched BM using Power SYBR® Green (Applied Biosystems) and 7500 Fast Real-Time PCR System, Applied Biosystems. Primer sequences are detailed in Additional file 1: Table S31.

## Additional files

Additional file 1:
**Lists of regulated genes in HR-MDS and AML post MDS relative to FVB/N (Tables S1 to S31).** (XLSX 1839 kb) Additional file 2: Figure S1. GSEA analysis, (A) Endplot of significantly dysregulated pathways in HR-MDS vs FVB/N, (B) Endplot of significantly dysregulated pathways in AML post MDS vs FVB/N (ZIP 5853 kb) Additional file 3: Figure S2. In a preliminary work, mRAS-BCL2 (referred in this study as MRP8[NRASD12/ hBCL-2], AML post MDS mice) transgenic mice Sca1+ spleen cells genes were analyzed on Affimetrix 430A mouse arrays. Compared to normal FVB/N Sca1+ spleen cells, (A) DAVID analysis, (B) Principal component analysis (PCA), and hierarchical clustering, were performed with R software (http://www.R-project.org) and Partek Genomics Suite (http://www.partek.com). Each sphere represents a single GEP from a given transgenic mouse: mRas (referred in this study as MRP8NRASD12 transgenic mice), mBCL2 transgenic mice (referred in this study as MRP8hBCL-2 transgenic mice), mRAS-BCL2 transgenic mice (referred in this study as MRP8[NRASD12/ hBCL-2] or AML post MDS transgenic mice), (C) Gene spring single gene analysis identified significant up-regulation of ADA, KRTCAP2, SSR4, CKAP4 and ATPG3 genes in mRAS-BCL2 transgenic mice (referred in this study as MRP8[NRASD12/ hBCL-2] or AML post MDS transgenic mice). (D). In this study; ADA and KRTCAP2 were up dysregulated in MRP8hBCL2 (mBCL2), AML post MDS (mRAS-BCL2) and HR-MDS transgenic mice Sca1+ spleen cells; SSR4 in AML post MDS (mRAS-BCL2) and HR-MDS transgenic mice Sca1+ spleen cells and; CKAP4 and ATPG3 in AML post MDS (mRAS-BCL2) transgenic mice Sca1+ spleen cells. (TIF 30355 kb)
